# SLIME: robust, high-speed 3D microvascular mapping

**DOI:** 10.1038/s41598-018-37313-z

**Published:** 2019-01-29

**Authors:** Yehe Liu, Meredith C. G. Broberg, Michiko Watanabe, Andrew M. Rollins, Michael W. Jenkins

**Affiliations:** 1 Department of Biomedical Engineering, Case Western Reserve University, Cleveland, USA; 2 Department of Pediatrics, Case Western Reserve University, Cleveland, USA; 3 Division of Pediatric Critical Care, UH Rainbow Babies & Children’s Hospital, Cleveland, USA

## Abstract

Three dimensional (3D) microvascular imaging of cubic millimeter to centimeter size volumes often requires much time and expensive instruments. By combining optical clearing with a novel scatter-based optical coherence tomography (OCT) contrast agent, we have greatly extended OCT imaging depth in excised tissues while maintaining a simple and low cost approach that does not require in-depth OCT knowledge. The new method enables fast 3D microvascular mapping in large tissue volumes, providing a promising tool for investigating organ level microvascular abnormalities in large cohorts.

## Introduction

Abnormal microvascular organization is often a sign of disease. Because microvasculature is a 3D network structure, some of the phenotype abnormalities could only be identified with large 3D volumes. Therefore, there is an emerging need for high throughput, statistical, organ-level assessment of microvascular morphologies in large cohorts. In recent years, various 3D microvascular mapping methods (e.g., micro-CT^1^, microscopy with serial slicing^2,3^, confocal microscopy^4^) have provided valuable information about the structure and function in various tissues and disease states. Unfortunately, many of these methods are either complex, expensive, and/or time intensive. They are not optimal for applications or studies involving both large volumes and large sample populations (e.g., studies involving diverse phenotypes, drug screening). To overcome this problem, we developed a simple, fast and low cost 3D microvasculature mapping method, termed “scatter labeled imaging of microvasculature in excised tissue” (SLIME).

SLIME is based on optical coherence tomography (OCT)^5,6^. Conventional OCT has millimeters field of view (FOV), 1–2 millimeters imaging depth and ~10 µm resolution^6^. Most conventional OCT has a fast acquisition rate (multi-cubic-millimeters per minute) and low cost (<$50k) compared to other 3D imaging modalities such as confocal microscopy and micro-CT. Additionally, OCT is already widely used as a tool for *in vivo* angiography^7–9^. However, there are some limitations with current OCT angiography (OCTA) methods. Vascular contrast of OCTA is generated through signal variation caused by blood flow, which is often sensitive to bulk motion, and requires the samples to have active and connected circulation. Also, light scattering and shadowing artifacts limit OCTA imaging depth to <1 mm. Because of these limitations, OCTA use is confined to transparent tissues (e.g., retina) and anatomical regions near the surface (e.g., brain surface^8^), which limits its applications in other vascular-related studies (e.g., deep regions in the brain). Taking a different approach from OCTA, SLIME broadens the vascular imaging capability of OCT through a combination of a novel OCT vascular contrast agent and optical clearing. While SLIME is incapable of imaging living animals like OCTA, it still benefits from high speed, high resolution, and a wide FOV. Further, SLIME does not have shadowing artifacts that are common in OCTA^8^ and the imaging depth is much greater and only limited by the optics of the OCT system (~2 mm/single scan for capillary resolution, see supplemental documents for more details about image depth and resolution).

**Figure 1:**
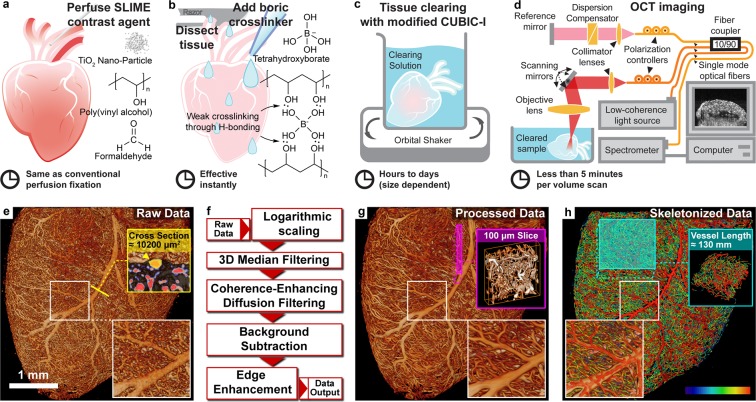
Overview of the SLIME procedure. (**a**) Perfusion of the animal with SLIME contrast agent. The SLIME contrast agent contains colloidal TiO_2_ nanoparticles, PVA, and paraformaldehyde. The perfusion procedure is identical to conventional perfusion fixation. (**b**) Contrast agent crosslinking through a PVA-boric acid slime reaction. The perfused organ is rinsed with a few drops of saturated boric acid and dissected out. The contrast agent near the surface of the tissue crosslinks rapidly through the slime reaction. Viscous slime is formed near the surface of the tissue, blocking the vessel exits and preventing leakage of the contrast agent. (**c**) Optical clearing. The sample is gently shaken in modified CUBIC-I clearing solution. The solution increases the transparency of the tissue, but the contrast agent remains highly scattering. The clearing solution also contains crosslinking agent that diffuses into the tissue with the detergent further stabilizing the contrast agent. (**d**) OCT imaging of the cleared SLIME sample. The panel demonstrates a conventional configuration of OCT. The sample is imaged in the optical clearing solution. (**e**) Volume rendering (see method for details) of the unprocessed SLIME data. SLIME raw data can readily be used for some simple analysis. The yellow line indicates the location of the subfigure in the yellow box, showing vessel cross-sections (labeled in red) and the calculated cross-sectional area of a large vessel (pointed by the yellow arrowhead). (**f**) SLIME data processing pipeline, indicating the important steps of SLIME data processing (**g**) SLIME data after processing. The magenta box demonstrates a 100 × 1000 × 1000 µm sub-volume with volume rendering. (**h**) Skeletonized SLIME data. The color bar indicates the relative vessel diameter (smaller in blue/larger in red). The cyan box shows a skeletonized sub-volume that has a total vessel length around 130 mm. The subfigures in the white boxes are 2x magnified views of the labeled locations. Examples of the procedure are also shown in Supplemental Figs 1 and 2. Supplemental Video 1 shows a volume rendering of a stage 36 quail embryo heart.

SLIME is not a contrast agent alone. It is an integrated method with tissue processing, imaging and data processing. Briefly, the vasculature is filled with a specialized OCT contrast agent to produce a strong OCT signal (Fig. 1, Supplemental Figs 1 and 2). The contrast agent is rapidly crosslinked to prevent leakage. The tissue is optically cleared to reduce light scattering and increase the accessible imaging depth. Because the contrast agent is not affected by optical clearing, the combined effect generates high OCT contrast between the filled vasculature and the rest of the tissue. Effective 3D image processing specific for SLIME allows visualization and quantification of microvascular morphology. The overall SLIME pipeline allows users to obtain 3D microvascular maps of small animal organs with minimal operation time.

The perfusable OCT contrast agent was formulated to optimize both its backscattering and viscosity properties. We identified 10% colloidal TiO_2_ nanoparticles (Aerosil Aeroxide P25, Evonik, anatase, primary particle size = 21 nm) as an ideal contrast agent for this application. 10% low molecular weight (M_w_ ~10,000) polyvinyl alcohol/acetate (PVA) is added as a dispersant, which also maintains the low viscosity of the solution needed for perfusion. Optionally, the solution can also include 4% paraformaldehyde (PFA) for longer preservation (Fig. 1a).

While perfusion requires low viscosity, keeping the contrast agent in the blood vessel requires low fluidity. Previous vascular contrast methods change temperature (e.g., gelatin gelation^10^) or use delayed polymerization (e.g., polymer resin^11^) to reduce fluidity. However, these methods impose time or temperature constraints, which are inconvenient for sample preparation involving large cohorts. Therefore, we adapted a different approach in SLIME. After perfusion, we treat the tissue with 4% boric acid, which crosslinks contacting PVA in a few seconds and creates a sticky non-Newtonian polymer that is usually referred as the toy “Slime”^12^. Slime clogs any vessel openings that have been created during the procedure, which effectively prevents contrast agent leakage (Fig. 1b).

After crosslinking, the tissue/organ is harvested from the animal and immersed in a modified CUBIC-I clearing agent^13^ containing boric acid. Optical clearing is simple and fast. As CUBIC-I treatment generates sufficient transparency for OCT imaging (Supplemental Fig. 3), this is the only step required for optical clearing. Meanwhile, borate ions in the clearing solution further stabilize the contrast agent inside the tissue. Time for optical clearing varies depending on the tissue size. For small samples like a stage 36 quail embryo heart (~4 × 4 × 2.5 mm), it takes overnight to clear the tissue with gentle shaking (Fig. 1c). Cleared SLIME samples can be imaged directly in the clearing solution with conventional OCT (Fig. 1d). The microvasculature organization is reflected through intensity contrast directly in a single scan. Even with a slow OCT system with 10 kHz line rate, it takes less than 2 minutes to acquire a 4 × 4 × 3 mm FOV (XYZ) at 4 × 4 × 6 um voxel sampling, which is sufficient to resolve the organization of the microvasculature in most tissues we have tested.

Because SLIME raw data provides high contrast images of blood vessels, simple automated image processing can be performed to compute useful information using tools that are readily available (Fig. 1e,f). A multi-iterative 3D median filter is applied to effectively reduce speckle noise. Coherence enhancing diffusion filtering^14^ is applied to further reduce noise and improve vessel continuity. Image contrast and intensity uniformity can be further improved by background subtraction and 3D unsharp filtering. In addition to qualitative visual interpretation (Fig. 1g & Supplementary Video 1), processed vascular data can be segmented through simple intensity thresholding and skeletonized into 3D graph data for quantitative analysis. Topological assessments (e.g., skeletonization^15^) can be readily performed with existing tools (e.g., Amira, Fig. 1h).

**Figure 2:**
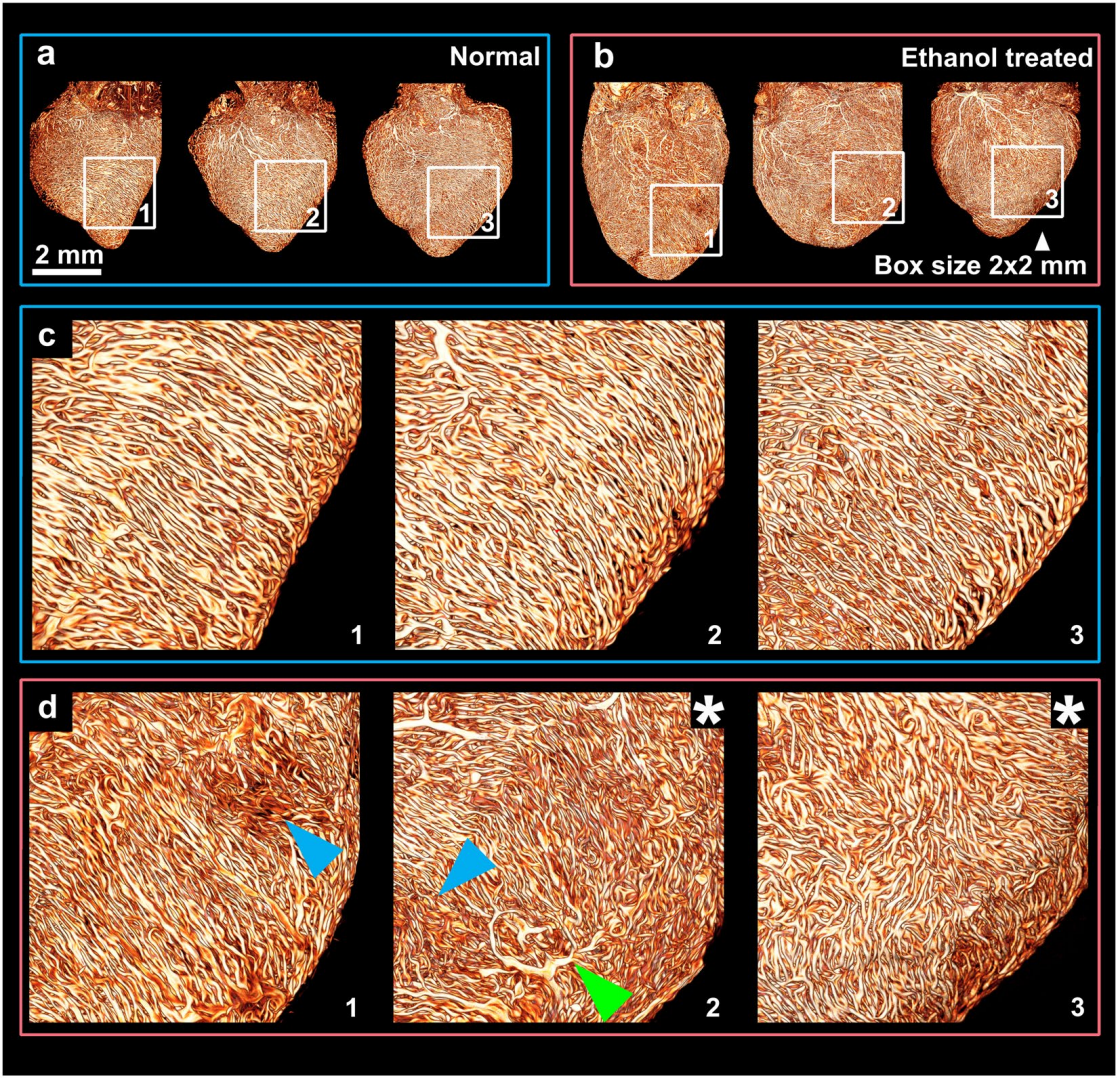
SLIME data from embryonic quails hearts exposed to alcohol. (**a**) SLIME images of 3 normal day 9 quail hearts. (**b**) SLIME images of 3 E9 quail hearts exposed to ethanol during gastrulation. (**c**) Close-up view of the regions in the white boxes in a, showing normal vascular patterns on the left ventricle. In normal ventricles, the coronaries near the surface are relatively parallel. (**d**) Close-up view of the regions in the white boxes in b, showing abnormal patterns. Blue arrows indicate regions with reduced vascular density. The green arrow indicates the abnormal occurrence of a large vessel on the surface. Asterisks indicates global chaotic patterns. Larger-sized and 3 additional images from each group are shown in Supplemental Fig. 4.

In some disease models, microvascular phenotypes at the organ level can vary significantly between individuals. Investigating a small number of samples and small portions of a sample can be inconclusive or misleading. However, with traditional imaging techniques (e.g., confocal microscopy), it is often resource intensive to acquire high resolution, large FOV images for a large population. This problem can be effectively addressed with SLIME, which is capable of mapping the microvasculature at the organ level with micrometers resolution and millimeters FOV within minutes. As a demonstration, we use SLIME to produce high-resolution data of quail embryo hearts in a well-established model of fetal alcohol syndrome (FAS) with diverse cardiovascular defects^16^ (Fig. 2, Supplemental Fig. 4). We identified various atypical microvascular phenotypes (e.g., chaotic alignment, reduced vessel density, abnormal organization). These microvascular abnormalities can be subclinical, but may eventually lead to more serious heart defects and disease. For instance, because microvasculature alignment is correlated with cardiomyocyte alignment, misaligned microvasculature may be associated with myocardial dysfunction^17^. More research is needed to determine how these abnormalities affect heart function over a lifetime.

**Figure 3:**
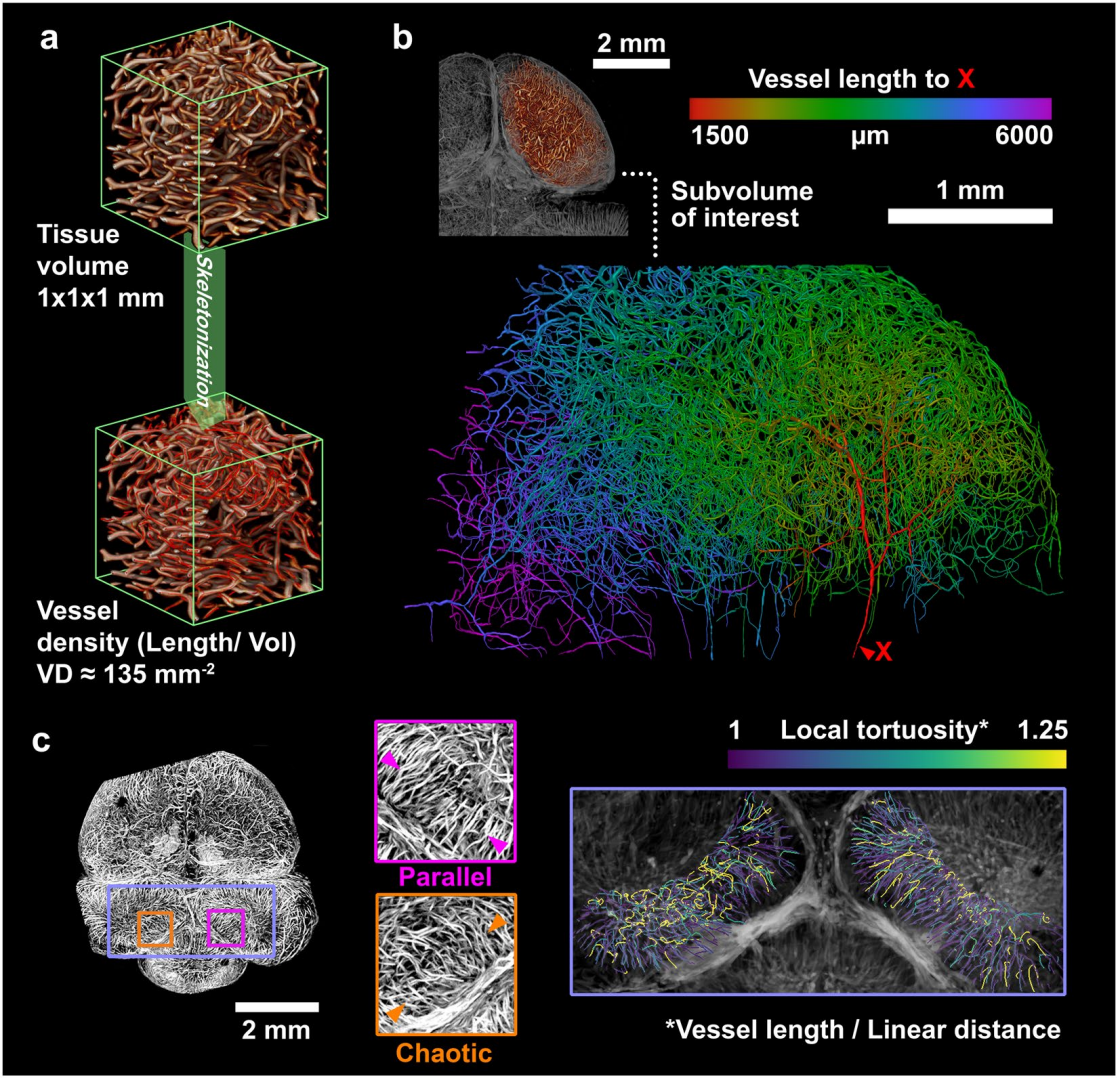
SLIME data from embryonic quails brains, demonstrating possible quantitative analysis. (**a**) Example vessel density (vessel length/unit volume) calculations from an arbitrarily selected 1 × 1 × 1 mm volume from the center of a normal E9 quail brain. More information about the vessel density measurement is shown in Supplemental Fig. 7. (**b**) Distance map showing the connected vessel length from near the beginning of the posterior cerebral artery. (**c**) Preliminary SLIME data indicates potential abnormalities in an E9 quail brain after ethanol exposure. The image on the left is a maximum intensity projection rendered from a 1 mm thick section in the middle of the SLIME volume, with colored boxes indicating close-up views of local regions. Symmetrical anatomical regions (left/right cerebellar hemispheres) showing distinct vascular patterns (middle) and local tortuosity (right), potentially due to ethanol exposure.

Quantifying aspects of vascular morphology across an entire organ can generate numerous insights about blood perfusion and metabolism in both normal and disease states. Because of the high contrast of SLIME images and the ability to skeletonize the data, various quantitative measurements can be easily made. As a proof of concept, we demonstrated the quantification of SLIME images for a stage 36 quail embryo brain (Supplemental Video 2). We show that vessel length density (total vessel length/tissue volume (Fig. 3a, Supplemental Fig. 5) and distance maps indicating vessel length to specific points of interest can be readily calculated from SLIME data using existing tools (Fig. 3b, Supplemental Video 3). We further demonstrate that quantitative measurements of SLIME images can be used as metrics to assess abnormalities. Normal embryonic quail brains show symmetrical vascular patterning between the left and right side. In the brains of the quail FAS model, SLIME data showed clear differences in local tortuosity between the left and right optical lobes (Fig. 3c and Supplemental Fig. 6).

In summary, for researchers who are interested in organ level microvascular organization, SLIME is a convenient and cost effective method for evaluating microvascular morphology over a large FOV in large cohort studies in a variety of animal tissues (including those of rodents, Supplementary Videos 4–6). Previously, OCTA provided a powerful tool for *in vivo* microvascular imaging that helped many vascular related studies. Now, as a compliment to OCTA, SLIME further expands the usefulness of OCT in vascular related studies, providing a promising tool for current OCTA users with almost no extra cost. With readily available image processing tools (e.g., ImageJ^18,19^, Amira), SLIME is able to provide quantitative information about 3D vasculature organization, which can be directly used for statistical analysis. It is worth mentioning that SLIME is a perfusion based method that only works on connected vessels, but on the other hand, SLIME can be applied to any biological vessels or lumens that can be perfused (e.g., lymph tubes, digestive tract). In addition, the PVA slime reaction can also potentially be adapted for delivering other colloidal-based contrast agents for other imaging modalities.

## Method

### SLIME Contrast Agent

The contrast agent stock solution is a mixture of 12.5% w/w titanium dioxide (TiO_2_) nanoparticles, 12.5% w/w polyvinyl alcohol/acetate (PVA), 0.5% w/w glycerol and 2.5% w/w commercial paint wetting agent in water. The concentration of each ingredient can be adjusted to achieve different image contrast and solution viscosity. To prevent aggregation of TiO_2_ nanoparticles in PVA solution, concentrated TiO_2_ water colloid and PVA solution are prepared separately and mixed to make the final colloid solution just before use. A commercial water-based paint wetting agent (Solsperse 27000, Lubrizol) is diluted before adding TiO_2_ nanoparticles (Aerosil Aeroxide P25, Evonik, anatase, primary particle size = 21 nm) to make a 25% w/w colloid solution. For each gram of TiO_2_ nanoparticles, 200 mg of wetting agent is used. After gentle stirring using a magnetic stirrer, the mixture is sonicated using a 250 W, 20 kHz, ultrasound homogenizer. Sonication at full power for 1 minute is sufficient to temporarily disperse 25 g of TiO_2_ nanoparticles in 5 g of wetting agent dissolved in 70 g of water. Low molecular weight PVA (M_w_ 9000–10000, Sigma, 80% hydrolyzed) is dissolved in deionized water at 80 °C under constant stirring to make a 25% w/w solution. The 25% TiO_2_ colloid is mixed 1:1 with the 25% PVA solution at 80 °C. 0.5% w/w glycerol is added to the mixture as a plasticizer. The mixture is further sonicated for 1 min per 100 g of mixture. The final colloidal solution should be very stable and have a shelf life of more than 3 months. If small amounts of precipitation occur during storage, sonication can be used to re-disperse the constituents. The stock solution is diluted 4:1 in 5X phosphate buffered saline (PBS) or 20% paraformaldehyde (PFA) to make a working solution.

### Crosslinking Solution

4% w/v boric acid is the crosslinking solution. The pH of the solution is titrated to 7.8 with 1 N sodium hydroxide. The solution should be stored at 37 °C. Precipitation gradually occurs when the temperature is lower than the storage temperature, but will not significantly affect the performance of crosslinking. 0.05% w/v NaN_3_ may be added as a microbicide for longer storage time.

### Clearing Solution

The clearing solution is modified from the Scale CUBIC-1 clearing agent^13^, which contains 25% w/w urea, 25% w/w Quadrol, 15% w/w Triton-X100, 15% w/w crosslinking solution and 15% w/w water. 25% w/w glycerol can be used as an alternative to Quadrol. To prepare 100 g clearing solution, 25 g Quadrol and 15 g crosslinking solution is dissolved in 15 mL DI water with continuous stirring using a magnetic stirrer hotplate. Heating or 20 kHz sonication can be applied to accelerate the solubilization, but the temperature of the solution should be maintained below ~50 °C. After the solution is completely mixed, 25 g of urea is added and dissolved by continuous stirring. The solution is cooled to room temperature and weighed. To compensate for the evaporation during the previous steps, DI water is added to make the total mass of the solution 85 g. Then 15 g of Triton X-100 is added and stirred slowly at room temperature until completely mixed. The solution can be stored at room temperature for up to 3 months.

### Perfusion

Prior to perfusion, a working solution is made by mixing the SLIME contrast agent stock solution 4:1 with 5X PBS or 20% PFA. The mixture is gently shaken for 5 minutes with a nutating mixer. Vigorous shaking should be avoided to prevent air bubble formation. In general, the perfusion protocol is identical to generic perfusion fixation protocols^20^ except the fixative solution is replaced with the SLIME contrast agent. Several small modifications to the SLIME perfusion protocol are needed to accommodate different animal models and tissues of interests. For embryos from smaller animals (e.g., mouse, avian), the animal can be perfused directly with the working solution. For larger animals (e.g., adult mouse and rat), the vasculature should be first flushed with anticoagulation solutions such as heparinized saline. The left ventricle is a convenient cannulation site for SLIME perfusion. Alternatively, if intact coronary circulation is being studied, perfusion can be done at the aortic arch or abdominal aorta. Because the contrast agent is white, the endpoint of perfusion is easily observed at low magnification. The tissues of interest turn pale and the large vessels become distinctly white. After perfusion, the tissues of interest are rinsed using the borate crosslinking solution. For tissues buried under other structures (e.g., brain under the skull), the crosslinking solution can be injected into the interfaces and cavities near the tissue using a syringe. After a brief waiting period of about 30 s, the tissues can be dissected out of the body using appropriate tools and procedures. As the tissues are surrounded by the crosslinking agent, the contrast agent rapidly turn into a viscous and sticky material which occludes vessels once the dissection takes place. If the vessels are pressurized or crosslinking is insufficient, leaking of contrast agent may occur. Adding more crosslinking agent with gentle compression can effectively stop the leakage. If contrast agent collects on the sample surface and impedes sample handling (e.g., sticks to the instruments), the contrast agent on the sample surface can be hardened and peeled off with a brief rinse of formamide.

### Optical clearing

After dissection, samples are cleared by immersing in excess modified *Scale*CUBIC-I solution. Clearing time depends on the size of the sample with bigger samples taking significantly longer to clear. For instance, a stage 36 quail embryo heart (~2.5 × 4 × 4 mm) only requires about 12 hours to clear. The brain from the same stage quail embryo (~3.5 × 8 × 8 mm) requires more than 24 hours to clear. It can take more than 5 days to clear the brain of a P5 mouse (~6 × 12 × 12 mm). To reduce the time for clearing large tissue samples, the samples can be dissected into ~2 mm thin slices using a vibratome, which reduces the clearing time to less than 24 hours. For faster clearing speed, the clearing solution can be replaced with fresh solution every 2 hours.

### OCT imaging

The cleared SLIME sample can be directly imaged using conventional OCT. The sample is immersed in optical clearing solution during OCT imaging. To eliminate reflection from the curved liquid-air interface, a microscope coverslip is placed on top of the sample. Because the light is polarized, signal from the cleared tissue can be suppressed by cross-polarizing the light between the sample arm and the reference arm (Supplemental Fig. 7). In contrast, TiO_2_ nanoparticle clusters depolarize the light, and therefore are not strongly affected by the cross-polarization. Since the intensity ratio between the contrast agent and the cleared tissue is high with this simple setup, sufficient image contrast can be obtained with just a single volume scan. It is worth mentioning that conventional OCT with Gaussian focusing only has the optimal lateral resolution in the Rayleigh range^6^. As focal power increases, resolution increases and depth of focus decreases. With conventional OCT, both large axial range and high resolution can be achieved through volume stitching. In this case, multiple volume scans should be taken by focusing the beam at different depths of the tissue (Supplemental Fig. 8). Alternatively, OCT with Bessel beam illumination could be used with SLIME to achieve both high resolution and extended depth in single scans^21^.

### Data Processing and analysis

Data is processed using customized code (code available on request) and image processing software. Conventional OCT processing (e.g., wavenumber linearization, Fourier transform) is performed on SLIME raw data to make typical OCT structure images. For higher image contrast, log compression can be omitted. If necessary, regions of interest are manually segmented to minimize processing time before further image processing. Some quantitative data analysis, such as cross-sectional area and circumference of large vessels can be performed directly without further image processing. To perform vessel cross-sectional area or circumference calculations, orthogonal slices to a vessel of interest are selected and the area or circumference are manually segmented and computed with Fiji (ImageJ)^18,19^.

Additional processing of the SLIME images is performed to reduce noise and improve data interpretability after log compression. First, 3 iterations of a 3D median filter are applied to reduce noise. Next, the data are filtered with a 3D coherence-enhancing diffusion filter to remove gaps in the vessel caused by OCT speckle noise^14^. These filters are anisotropic and smooth along the length of linear structures (e.g., vessels) which effectively reduces speckle noise and enhances vessel connectivity which improves the ease of visual interpretation and automated vessel segmentation. Occasionally, samples are not sufficiently cleared and background subtraction is used. In this situation, background is estimated with a down-sampled, blurred image. To further improve vessel contrast, the data are processed using either an unsharp mask or high pass filter.

In this work, data skeletonization is performed with Amira (Thermo Fisher Scientific). One of the most frequently used visualization methods demonstrated in this manuscript is referred to as “volume rendering” (e.g., Fig. 1e). It is a ray tracing based rendering technique that simulates the voxels of 3D objects as solid matter. Different voxel values are mapped to different optical properties. Usually larger voxel values are simulated as more solid and scattering materials, while smaller values are more transparent. A simulated light source casts rays onto the objects, creating reflections, diffusions and shadows to form an image. Volume rendering is most suitable for studying vessel morphology near the surface. For visualizing internal structures deep in the volume, the sample can be cropped/clipped into smaller/thinner volumes.

To get a binary map of the vasculature for skeletonization^15^, we used simple intensity-based thresholding. With skeletonized data, local lengths, locations and orientations of local vessel segments can be evaluated and used to calculate other parameters such as vessel length density (Supplemental Fig. 7) and local tortuosity (Supplemental Fig. 8)^22^. Here, vessel length density is defined and calculated by counting the total length of the vessel in a unit volume of tissue. Because vessel diameter can vary significant due to preparation and measurement errors, we believe vessel length density is a better measurement of vessel density compared to volume fraction.

### Animals

According to IACUC guidelines at Case Western Reserve University, the policy on the use of Avian Embryos states that, “If embryos will be sacrificed prior to 3 days before hatching, the research will not be subject to IACUC review.” Domestic quail embryos typically hatch around embryonic day 17. Therefore, IACUC approval was not required for quail embryos in this study. Fresh fertilized quail eggs were purchased from a commercial supplier. The eggs were incubated in an egg incubator at 37 °C with near saturated humidity, until they achieved the desired developmental stages.

For the preliminary tests on rodents, all procedures were conducted in accordance with Case Western Reserve University IACUC-approved protocols. All rodents were leftover wild type animals from unrelated studies. They were euthanized by perfusion fixation under anesthesia prior to SLIME perfusion.

### Quail fetal alcohol syndrome (FAS) model

The primary reason to use the quail FAS animal model was to help validate the technique. The FAS model was easy to use and causes a large variety of cardiovascular structural abnormalities at different scale levels^16,23,24^. We hypothesized that some abnormalities could also occur at the microvascular level, and would be diverse in their presentation. The goal of the work was to demonstrate SLIME’s ability to identify microvascular abnormalities that may be missed using traditional techniques. A full study using SLIME to assess the microvasculature will be done in the future.

To generate quail embryos with FAS, we followed a well-established protocol used in our previous studies^16,23,24^. In brief, quail eggs were incubated at 37 °C until gastrulation (~21 hours). The eggs were then injected with 40 µl of 50% ethanol in normal saline solution. The injection sites were sealed, and the eggs were kept in the incubator until they reached the desired developmental stages.

To validate SLIME’s ability to capture microvascular phenotypes across an entire organ, we compared the SLIME results of 6 normal quail embryo hearts and 6 quail embryo hearts from the FAS model. The coronary patterns from the SLIME data were evaluated by a human expert.

To demonstrate SLIME’s ability to make quantitative measurements, we also examined some stage 36 quail embryo brains from both groups. Because the brains at this stage of development have large volumes, diverse vascular morphology and high structural symmetry without being too dense and overlapping, they were ideal for validating and demonstrating quantitative assessment. We performed vessel length density, length to specific points and local tortuosity measurements. Using the symmetry of the brain, the measurements were validated by comparing analysis made on both sides of the brain.

## Supplementary information

Supplemental Video 1

Supplemental Video 2

Supplemental Video 3

Supplemental Video 4

Supplemental Video 5

Supplemental Video 6

Supplemental documents
