# Supplementary material for: SLIME: robust, high-speed 3D microvascular mapping
Source: Sci Rep. 2019 Jan 29;9:893. doi: 10.1038/s41598-018-37313-z (PMC6351571; doi:10.1038/s41598-018-37313-z)
Supplement: Supplementary file 7 — Supplemental documents [file 41598_2018_37313_MOESM7_ESM.docx]

**SLIME: robust, high-speed 3D microvascular mapping**

Yehe Liu^1^, Meredith C. G. Broberg^2,3^, Michiko Watanabe^2^, Andrew M Rollins^1^, Michael W Jenkins^1,2^

^1^Department of Biomedical Engineering, Case Western Reserve University

^2^Department of Pediatrics, Case Western Reserve University

^3^Division of Pediatric Critical Care, UH Rainbow Babies & Children’s Hospital

**Supplemental Figures**


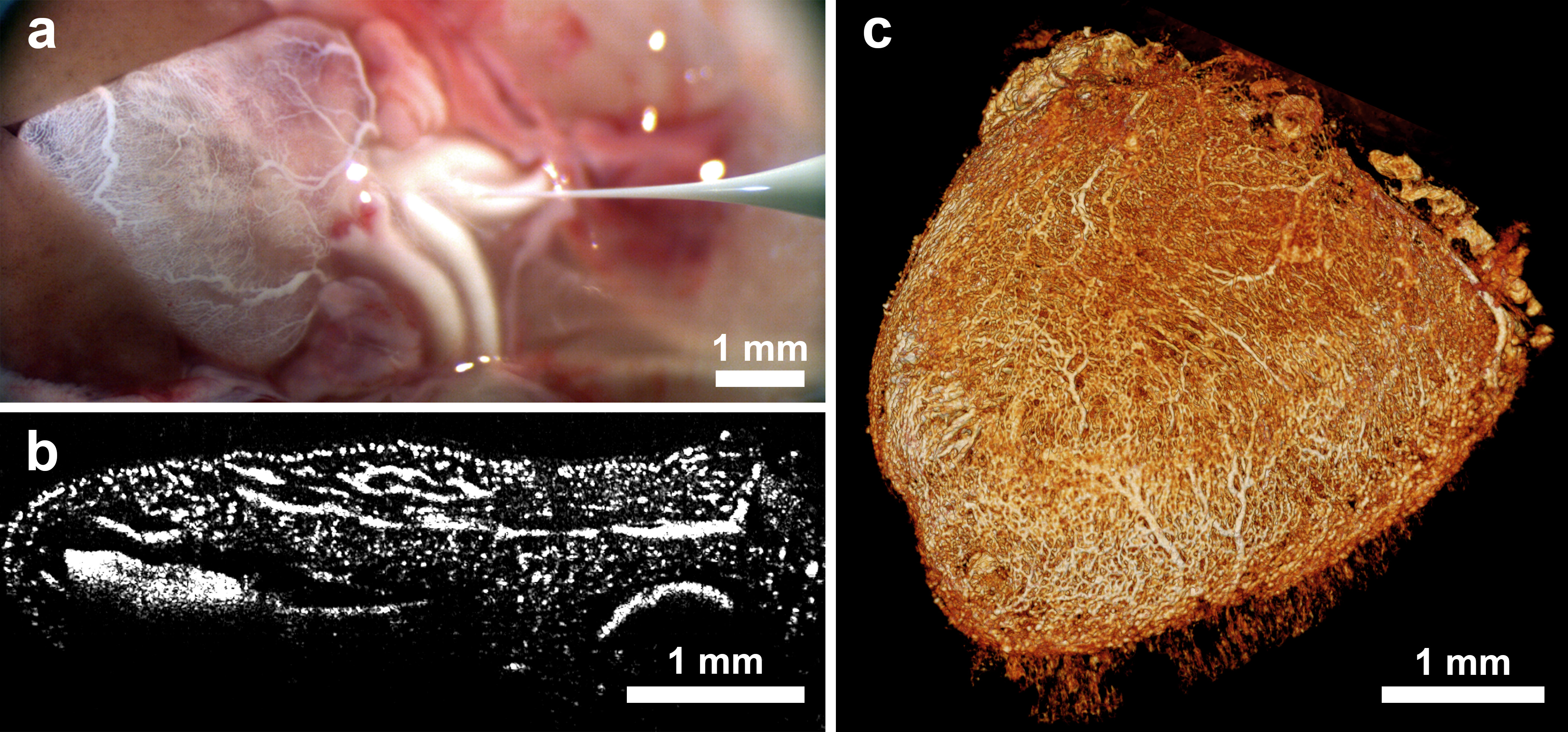


Supplemental Figure 1 | a) Stereoscopic image of SLIME contrast agent perfusion in an E9 quail embryo. b) Typical OCT B-scan (frame scan) showing a single cross- sectional area of the E9 quail embryo heart treated with SLIME. c) SLIME raw data volume rendering of an E9 quail embryo heart.


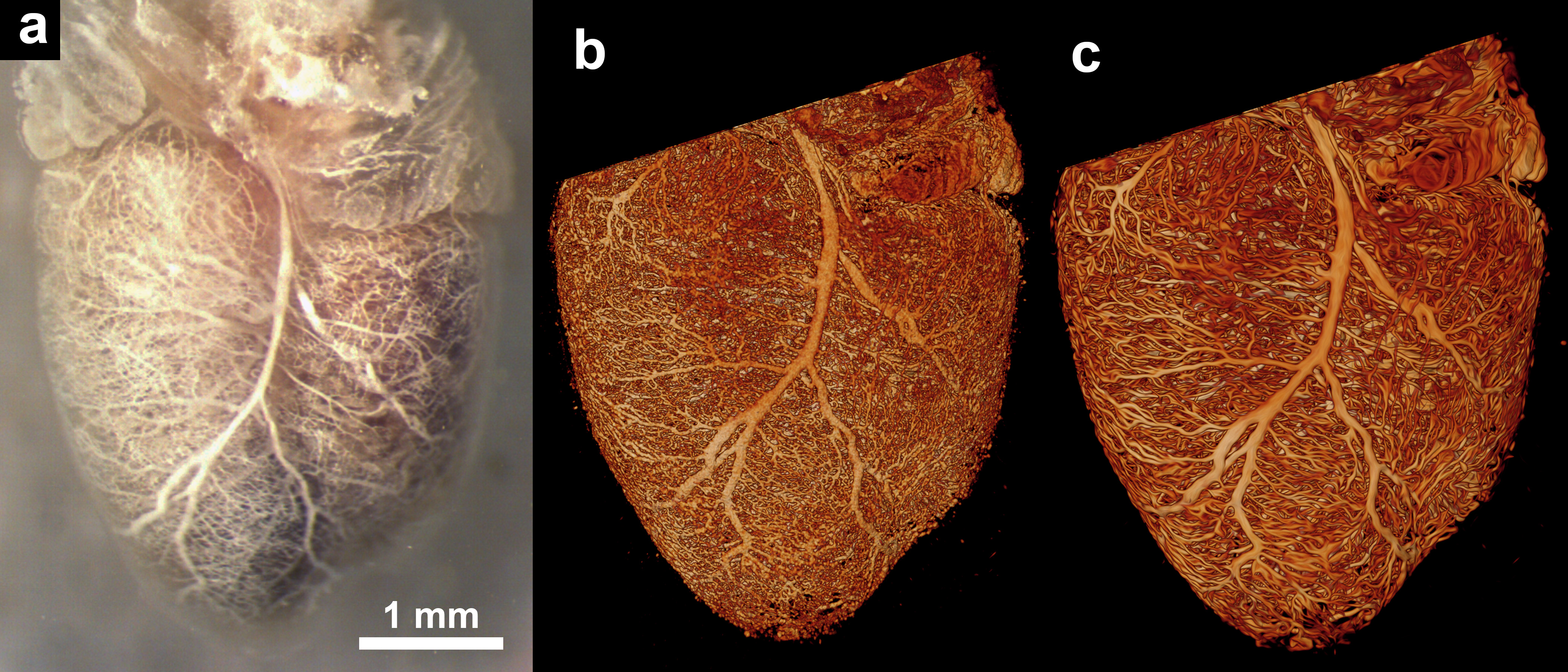


Supplemental Figure 2 | Comparison among a) steromicroscopic image, b) volume rendering of raw SLIME data and c) volume rendering the processed SLIME data of an E9 quail embryo heart (back side). SLIME accurately reflects the morphology of the vessels.


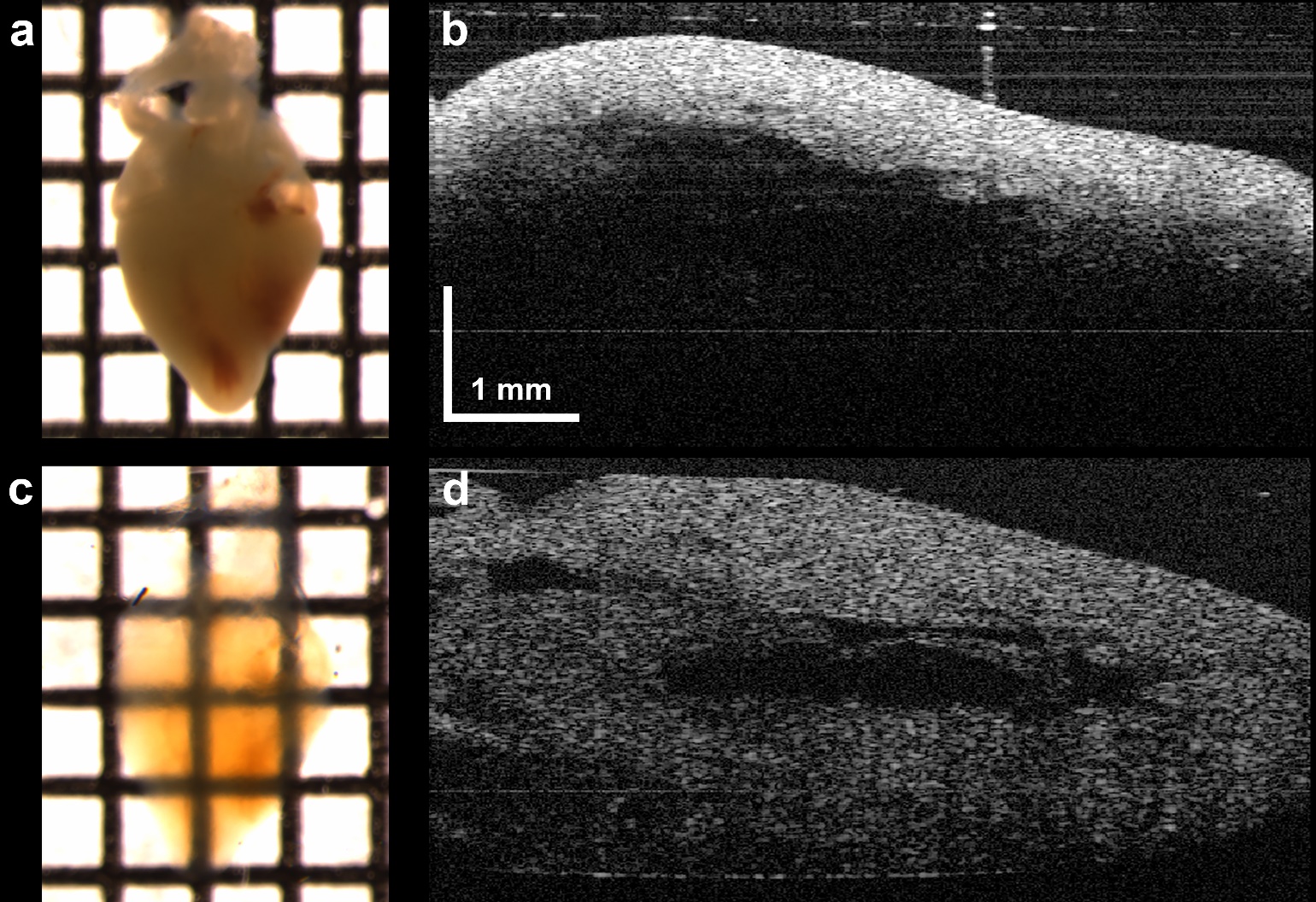


Supplemental Figure 3 | Effect of optical clearing on quail embryonic heart tissue. a) Stereomicroscopic image of a fixed E8 quail embryo heart in PBS (transmission illumination; grid size is 1x1 mm). b) OCT b-scan image of the same heart in a. c) Stereomicroscopic image of the same E8 quail embryo heart after being cleared in CUBIC-I solution overnight. d) OCT b-scan image of the same heart in c. OCT images in b & d were taken under identical conditions and rendered over the same dynamic range. Because light scattering is significantly suppressed by optical clearing, OCT signal from back scattering of the tissue is reduced, while imaging depth is drastically increased.


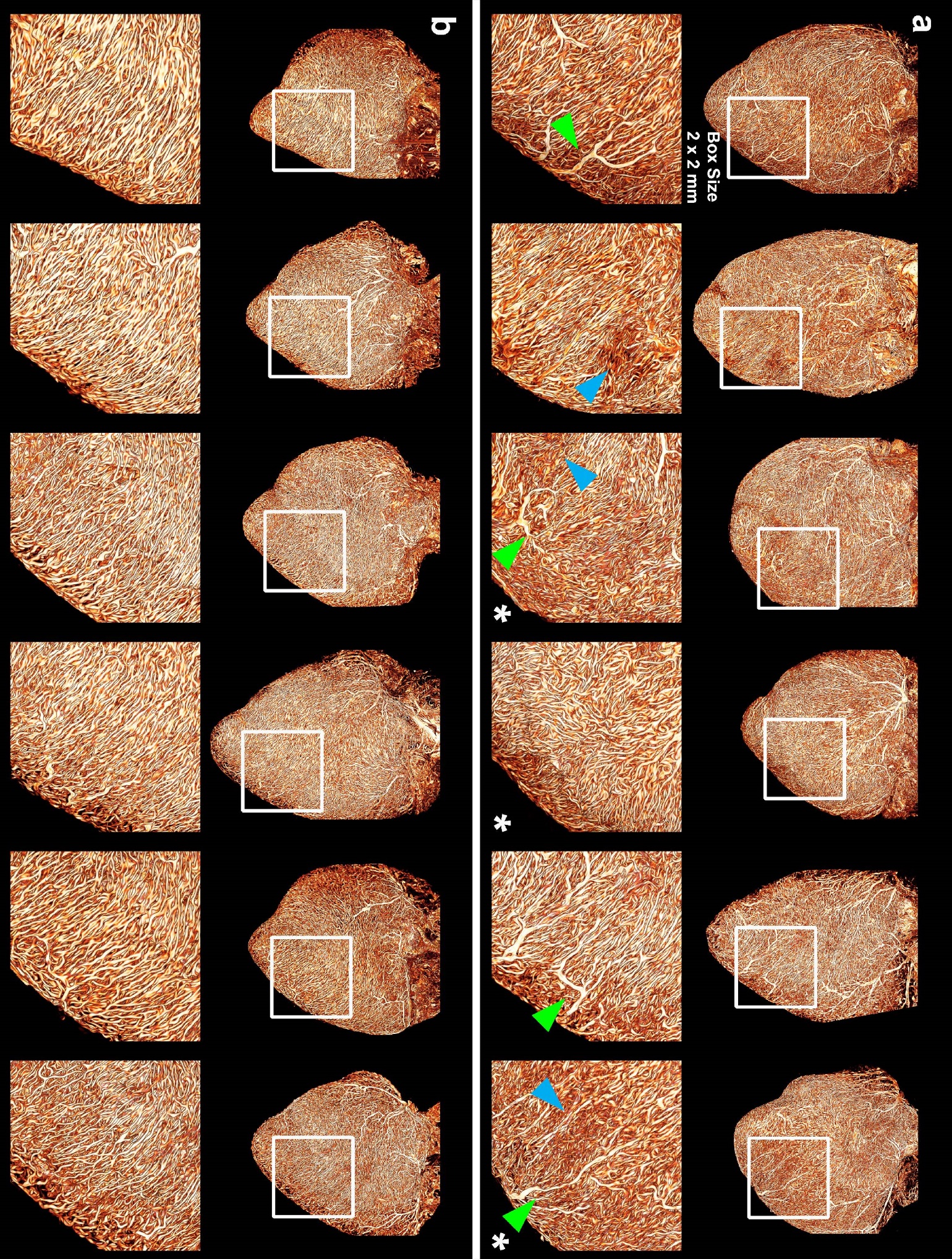


Supplemental Figure 4 | Higher resolution and additional prenatal alcohol exposure data a) SLIME images of 6 E9 quail embryo hearts from the ethanol treated group (front view). Top: global picture. Bottom: close up of the left ventricle labeled in the white boxes. Regions with reduced perfusion are indicated by the blue arrows. Abnormal location of large arterioles on the surface indicated by the green arrows. Global chaotic microvascular pattern indicated by the asterisks. b) SLIME images of 6 normal E9 quail embryo hearts for comparison.


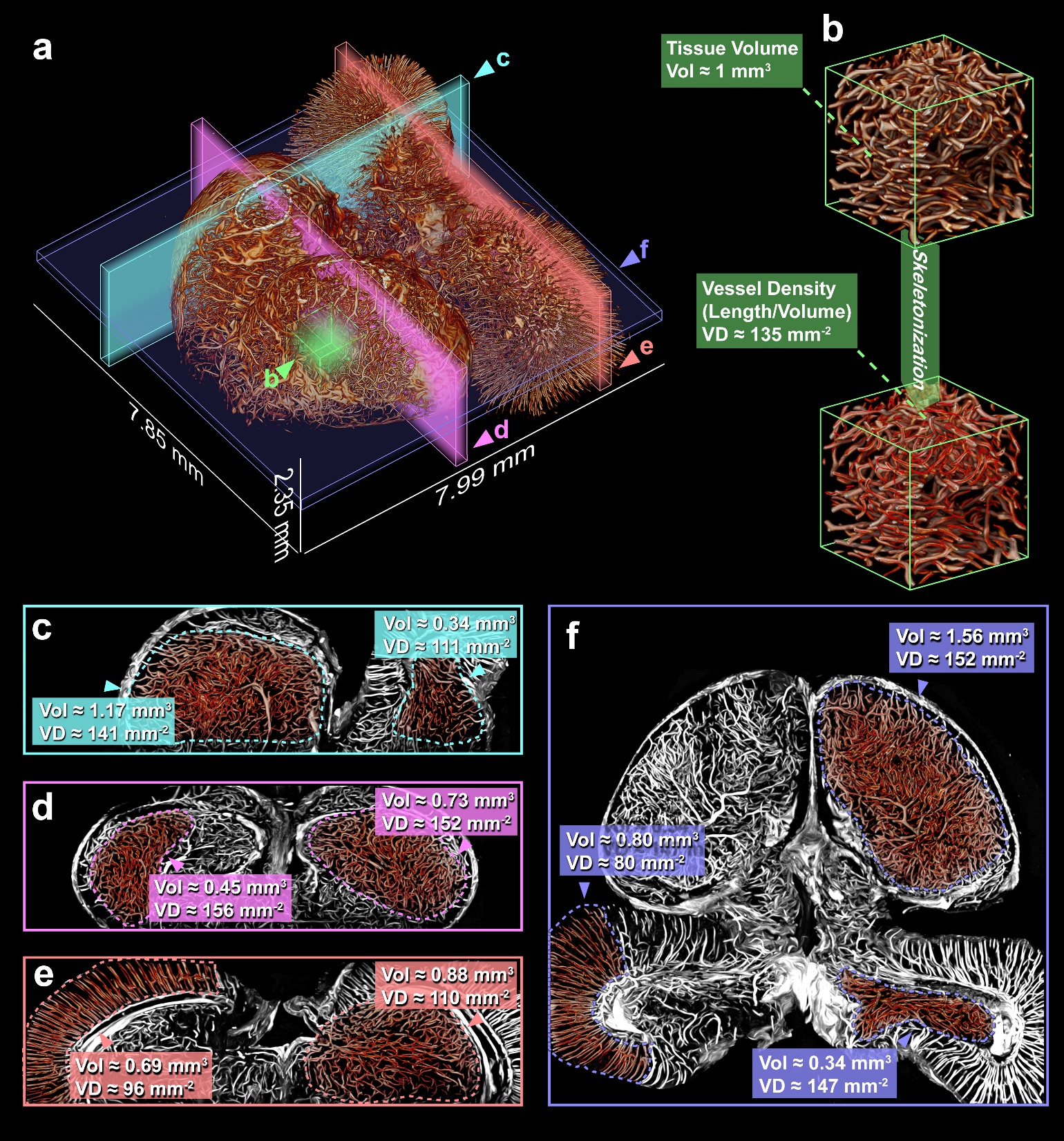


Supplemental Figure 5 | Vessel density (VD) analysis with skeletonized SLIME data. a) Volume rendering of a SLIME treated E9 quail embryo brain. Regions in b-f are labeled in color-coded boxes. b) Measurement of vessel density in selected tissue volumes. The volume of interest is user defined (randomly selected here) and calculated by voxel counting. The selected volume is skeletonized and the total length of the vessel is calculated based on the graph data. VD is defined as the total length of vessel in a tissue volume of interest divided by the volume size. c-f) calculation of VD in selected regions of the brain.


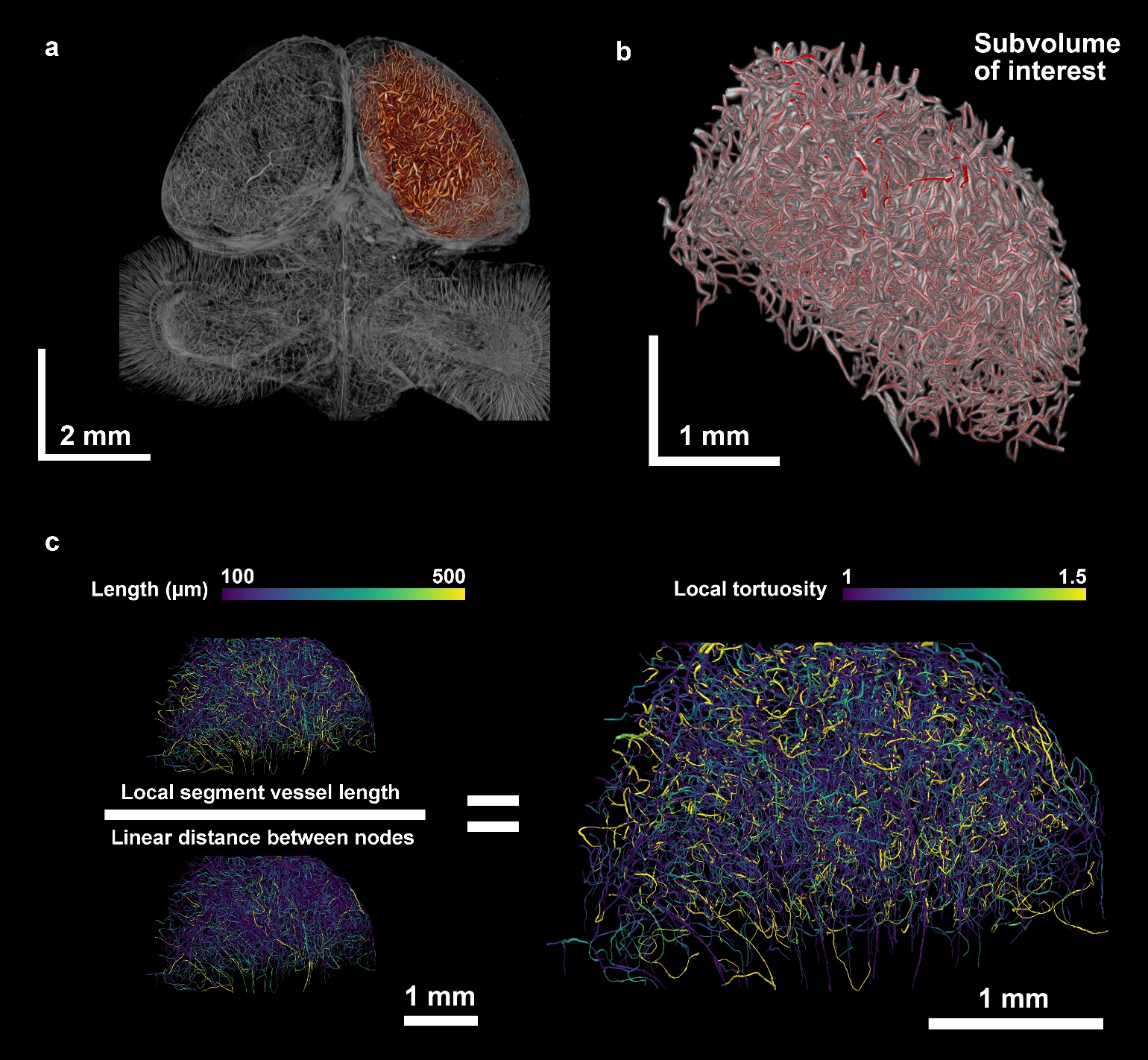


Supplemental Figure 6 | Local tortuosity analysis with skeletonized SLIME data. a) Volume rendering of a SLIME treated E9 quail embryo brain rendered in gray, with the subvolume of interest (right cerebral hemisphere) rendered in color. b) The subvolume of interest rendered in gray overlapped with red indicating the skeletonization. c) Calculation of local tortuosity by dividing the vessel length of local vessel segments with the linear distance between the end points of these segments.


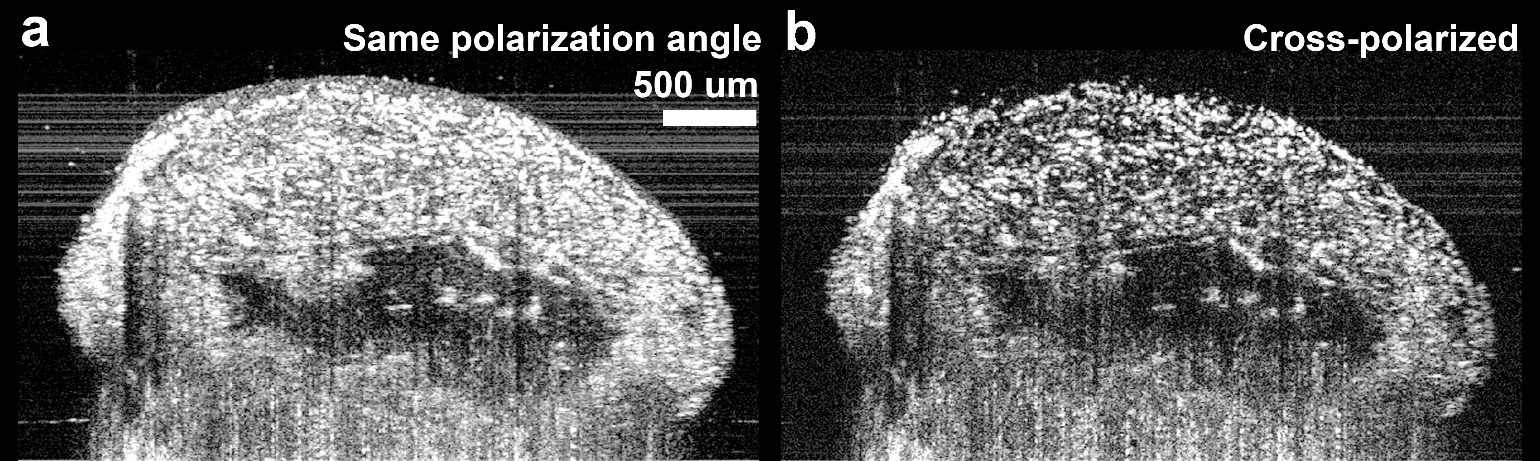


Supplemental Figure 7 | Effect of polarization on the SLIME OCT data. a) When polarization of the sample matches the reference arm, back scattering from the cleared tissue is not completely eliminated. b) By adjusting the polarization to be orthogonal to the reference arm, light scattering from the tissue is significantly reduced, while the back scattering from the contrast agent is minimally affected. This minor change to the standard optical setup reduces the work load for post processing of the SLIME data.


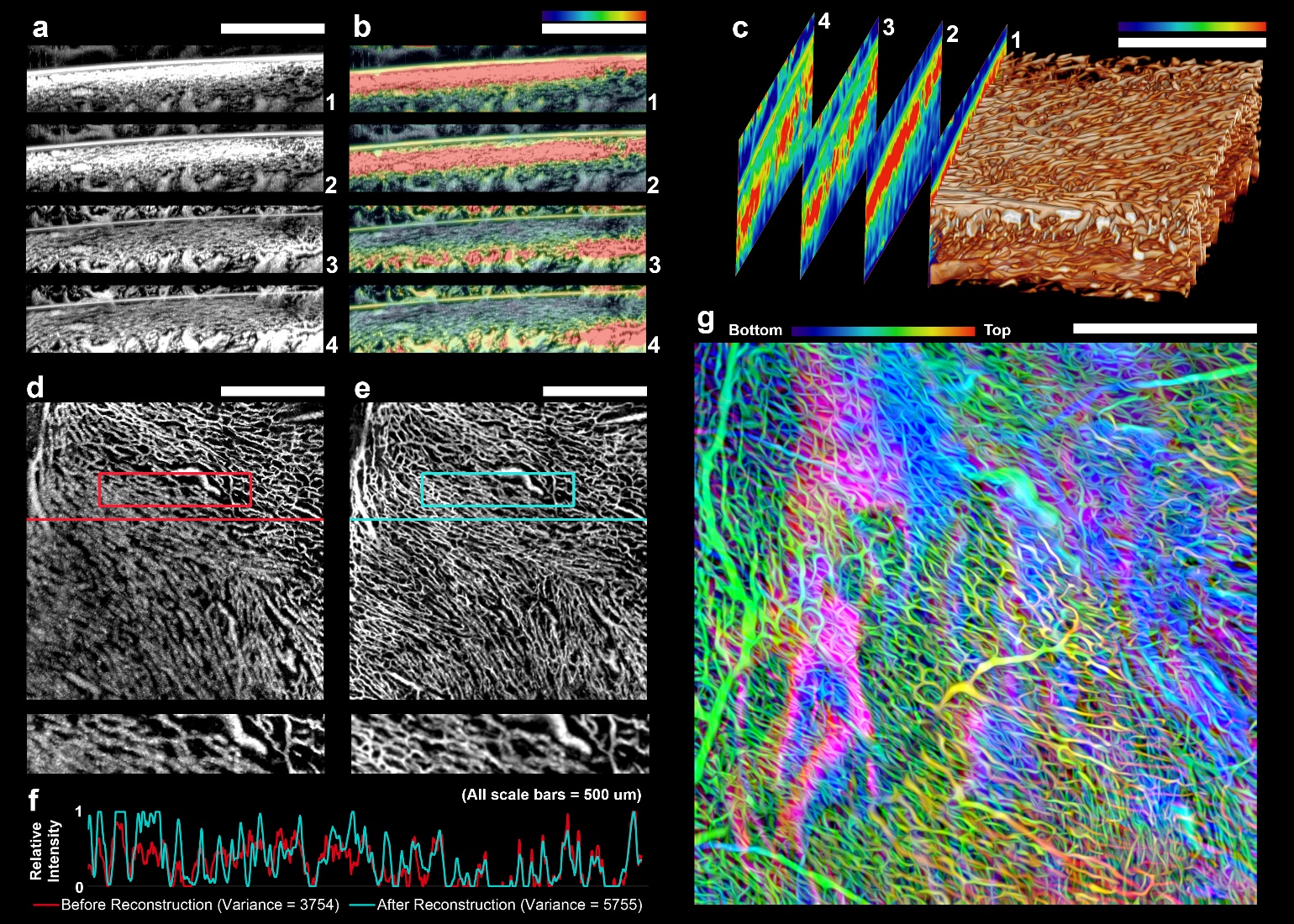


Supplemental Figure 8 | High resolution OCM images of coronary microvasculature from an adult mouse left ventricle constructed from four volumes focused at different depths. a) 50 µm side view maximum intensity projections of the 4 different volumes taken by moving the focus subsequently deeper into the tissue. Each volume was rigid-registered to the initial volume using a resolution degraded image. b) A local variance measurement selected of in-focus regions from each volume. Regions in focus usually have higher local variance than the out of the focused regions. The colormap represents relative local variance in hue. Warmer colors indicate higher local variance and higher resolution. c) Volume rendering of the reconstructed volume with representative slices showing corresponding local variances in each of the four volumes. Volume reconstruction is performed by selecting the voxels with the highest local variances from the 4 raw data volumes. d-e) Comparison of 10 µm maximum intensity projections near the middle of the volume. d uses one volume to construct the image, while e uses all four images. The small images below represent the regions labeled with the colored box on the top master images. Before reconstruction, only a large portion of the microvasculature is out of focus. After reconstruction, all of the microvasculature in the slice are well resolved. (Note: The objective was slightly tilted to prevent surface reflection so the focal plane is not horizontal.) f) Intensity profile (indicated by the colored line) on subfigure d-e showing higher intensity variation in the reconstructed volume. g) Color-encoded depth intensity projection showing microvascular organization in the entire volume.

**Additional Comments**

**Considerations for resolution and imaging depth:**

(Note: a few terminologies can be confusing in the following paragraph. “Imaging depth” means the maximum achievable axial depth. “Focal depth” means the location of the center focal point in the sample. “Depth of focus” or “focal range” means the axial range near the focus with acceptable resolution.)

In spectral-domain OCT systems, a single M-scan instantaneously resolves the structure in the axial direction at a resolution determined by the coherence length of the light source (l_C_) and the refractive index (n) of the sample:

$$\text{Axial Resolution} (\delta z)=\frac{\text{ }l_{C}}{n}=\frac{2\ln(2)\lambda_{0}^{2}}{\pi n\Delta\lambda\text{ }}$$

where λ_0_ is the center wavelength and Δλ is the full width at half maximum (FWHM) wavelength bandwidth (Izatt and Choma). The physical imaging depth is limited by spectral sampling of the system, which can be calculated by:

$$\text{Maximum Physical Depth} (L)=\frac{N ln(2)\lambda_{0}^{2}}{\pi n\Delta\lambda\text{ }}$$

where N is the number of spectral samples. For instance, a conventional OCT system using a 1310 nm ± 50 nm light source and sampling 2048 data points can provide 5.7 um axial resolution and 5.8 mm maximum imaging depth in water (n = 1.33).

In practice, axial image depth is also limited by the shape of the focused beam. Conventional OCT often utilizes Gaussian focusing where the lateral resolution is determined by the numerical aperture (NA) of the optical system:

$$\text{Lateral Resolution} \left( \delta x \right)=\frac{0.4\lambda_{0}}{NA}$$

As the beam radius increases away from the focal point, the lateral resolution degrades. The practical axial field of view can be estimated by the FWHM axial confocal response:

$$\text{Practical axial field of view} (L_{p})= \frac{0.57\lambda_{0}}{\sin^{2} (\sin^{-1} \left( NA \right)/2)}$$

For optically cleared samples (e.g., SLIME samples with low vessel density) with high optical transparency, it is easy to image through the entire sample so imaging depth and axial resolution are limited by the bandwidth of the light source and the spectral resolution of the interferometry. Although lateral resolution and focal depth have no direct effect on the axial resolution and imaging depth, it still limits the useful imaging depth if specific lateral resolution needs to be achieved. This causes a tradeoff between resolution and imaging depth. This tradeoff is shown in the figure below (assuming 1310 nm center wavelength):

In order to resolve individual vessels with SLIME, both axial and lateral resolution need to be greater than the spacing between adjacent vessels. This is on the order of 10 µm and depends on the tissue type. For tissues with low vessel density (e.g., brain and skeletal muscle), it is possible to use lower NA focusing to achieve longer imaging depths in a single scan (e.g., resolution = 16 um; depth = 2.5 mm; center wavelength = 1310 nm). For tissue with high vessel density (e.g., heart and kidney), imaging depth of a single scan is compromised because higher lateral resolution is often required (e.g., resolution = 10 um; depth = 1 mm; center wavelength = 1310 nm). If it is necessary to accurately measure the diameter of the capillaries, higher resolution setups such as optical coherence microscopy (OCM) can be used, but imaging depth is limited (e.g., resolution = 2.5 um; depth = 100 um; center wavelength = 800 nm).

Even though the field of view and image depth is compromised, SLIME with OCM still has advantages compared to conventional confocal microscopy with fluorescently labeled vessels. At the same galvo scan rate, OCM is much faster than confocal microscopy. Resolving microvasculature requires both sufficient axial and lateral resolution. Microscope objective lenses with low NA and low magnification usually can provide enough lateral resolution for vascular imaging. These objective lenses usually have relatively large fields of view and long working distances. These lenses are often inexpensive. However, they are insufficient for 3D confocal microvascular mapping due to poor axial resolution. Because OCM uses the coherent properties of light, OCM can use the low NA lenses to give sufficient axial and lateral resolution (e.g., OCM with 800 ± 75 nm light source can provide ~2 um axial resolution). In order to achieve high axial resolution using confocal microscopy, high NA objectives are required. These lenses are typically more expensive, have a smaller field of view (limited by magnification), and a reduced imaging depth (limited by working distance).

Two different approaches can help achieve long image depth and high imaging resolution. With conventional OCT, it is possible to take multiple volumes at different focal depths in the sample, then extract the regions with high lateral resolution, and reconstruct into a single image. As an example, we show reconstruction of OCM SLIME data from 4 continuous focal depths in supplemental Figure 8. This volume stitching concept can also be combined with serial sectioning to reconstruct images from larger tissue volumes, but it is necessary to automate the whole procedure to make it practical for high-throughput studies.

With advanced optical designs, both extended focal depth and high lateral resolution can be achieved at the same time. One approach is to use an electrical tunable lens to sweep the focus in the sample (Grulkowski et al.). This dynamic focusing setup can directly create high-resolution images with extended imaging depth. Another approach is to use Bessel beam illumination and detect the signal using low NA Gaussian focusing (Blatter et al.), which is also a well-established OCT imaging setup. These optical designs can be easily integrated into existing conventional OCT systems.

**Considerations for data analysis:**

As described earlier, although SLIME can provide sufficient resolution for resolving individual vessels, typical OCT resolution is not sufficient to resolve the fine morphology of local capillaries (e.g., diameter). Without using a high resolution system, measurement related to capillary diameter should be avoided. Data analysis should be focused on the global morphology and topology of the measured vasculature (e.g., length, number of branches, tortuosity, etc.). If detailed local morphology of the microvasculature is needed, high resolution systems such as OCM should be considered.

OCT images naturally contain speckles. The speckle size varies based on the specifications of the OCT system. Because blood vessels are connected linear network structures, coherence enhanced diffusion filtering can significantly reduce speckle noise based on the image features according to the original publication (Weickert). To further reduce speckle noise, optical-based speckle reduction methods can be used (Szkulmowski et al.).

Although optical clearing reduced scattering from the majority of the tissue, reflection still occurs on interfaces between structures with distinct optical properties (e.g., between the tissue surface and the surrounding optical clearing solution). Therefore, analysis on these regions should be avoided in the current version of SLIME. However, this problem can be remedied by avoiding imaging surfaces that are perpendicular to the focused beam, for instance, using Bessel beam illumination with low NA Gaussian detection.

**Considerations for sample type:**

SLIME is a perfusion based method. Therefore, it only labels connected tissue. In some pathological conditions, vessels can be blocked or closed. Although some free flowing contrast agent may diffuse into these disconnected vessel segments over time, SLIME is not an appropriate tool for identifying those disconnected vessels. Cell specific labeling technique should be employed instead (e.g., fluorescent immunohistochemistry).

It also worth mentioning that some disease models (e.g., cancer) contain disorganized and leaking vessels. When SLIME is applied to these samples, contrast agent may leak into the tissue and result in a large labeled region. On one hand, it can be difficult to resolve fine local microvascular structures due to the leakage. On the other hand, a measure of perfusion induced microvascular leakage can be potentially useful for related applications.

References

Blatter, Cedric, et al. “Extended Focus High-Speed Swept Source OCT with Self-Reconstructive Illumination.” *Optics Express*, vol. 19, no. 13, Optical Society of America, June 2011, p. 12141, doi:10.1364/OE.19.012141.

Grulkowski, Ireneusz, et al. “Swept Source Optical Coherence Tomography and Tunable Lens Technology for Comprehensive Imaging and Biometry of the Whole Eye.” *Optica*, vol. 5, no. 1, Optical Society of America, Jan. 2018, p. 52, doi:10.1364/OPTICA.5.000052.

Izatt, J. A., and M. A. Choma. “Theory of Optical Coherence Tomography.” *Optical Coherence Tomography, Biological and Medical Physics, Biomedical Engineering*, edited by Wolfgang Drexle and James G. Fujimoto, Springer, Berlin, Heidelberg, 2008, pp. 47–72, doi:10.1007/978-3-540-77550-8_2.

Szkulmowski, Maciej, et al. “Efficient Reduction of Speckle Noise in Optical Coherence Tomography.” *Optics Express*, vol. 20, no. 2, Optical Society of America, Jan. 2012, p. 1337, doi:10.1364/OE.20.001337.

Weickert, Joachim. “Coherence-Enhancing Diffusion Filtering.” *International Journal of Computer Vision*, vol. 31, no. 23, 1999, pp. 111–27, https://link.springer.com/content/pdf/10.1023/A:1008009714131.pdf.
